# Haemorrhagia post partum; an implementation study on the evidence-based guideline of the Dutch Society of Obstetrics and Gynaecology (NVOG) and the MOET (Managing Obstetric Emergencies and Trauma-course) instructions; the Fluxim study

**DOI:** 10.1186/1471-2393-10-5

**Published:** 2010-01-26

**Authors:** Mallory D Woiski, Rosella PMG Hermens, Johanna M Middeldorp, Jan A Kremer, Marco A Marcus, Maurice GAJ Wouters, Richard P Grol, Fred K Lotgering, Hubertina CJ Scheepers

**Affiliations:** 1 Department of Obstetrics and Gynaecology, Radboud University Nijmegen, Nijmegen, the Netherlands; 2 IQ Healthcare, Radboud University Nijmegen, Nijmegen, the Netherlands; 3 Department of Obstetrics, Leiden University Medical Centre, Leiden, the Netherlands; 4 Department of anaesthesiology, University Hospital Maastricht, Maastricht, the Netherlands; 5 Department of Obstetrics and Gynaecology, VU Medical Centre, Amsterdam, the Netherlands; 6 Department of Obstetrics and Gynaecology, University Hospital Maastricht, Maastricht, the Netherlands

## Abstract

**Background:**

One of the most important causes of maternal mortality and severe morbidity worldwide is post partum haemorrhage (PPH). Factors as substandard care are frequently reported in the international literature and there are similar reports in the Netherlands. The incidence of PPH in the Dutch population is 5% containing 10.000 women a year. The introduction of an evidence-based guideline on PPH by the Dutch society of Obstetrics and Gynaecology (NVOG) and the initiation of the MOET course (Managing Obstetrics Emergencies and Trauma) did not lead to a reduction of PPH. This implies the possibility of an incomplete implementation of both the NVOG guideline and MOET-instructions. Therefore, the aim of this study is to develop and test a tailored strategy to implement both the NVOG guideline and MOET-instructions

**Methods/Design:**

One step in the development procedure is to evaluate the implementation of the guideline and MOET-instructions in the current care. Therefore measurement of the actual care will be performed in a representative sample of 20 hospitals. This will be done by prospective observation of the third stage of labour of 320 women with a high risk of PPH using quality indicators extracted from the NVOG guideline and MOET instructions. In the next step barriers and facilitators for guideline adherence will be analyzed by performance of semi structured interviews with 30 professionals and 10 patients, followed by a questionnaire study among all Dutch gynaecologists and midwives to quantify the barriers mentioned. Based on the outcomes, a tailored strategy to implement the NVOG guideline and MOET-instructions will be developed and tested in a feasibility study in 4 hospitals, including effect-, process- and cost evaluation.

**Discussion:**

This study will provide insight into current Dutch practice, in particular to what extent the PPH guidelines of the NVOG and the MOET-instructions have been implemented in the actual care, and into the barriers and facilitators regarding guideline adherence. The knowledge of the feasibility study regarding the effects and costs of the tailored strategy and the experiences of the users can be used in countries with a relatively high incidence of PPH.

**Trial Registration:**

ClinicTrials.gov NCT00928863

## Background

The major cause of maternal death worldwide is post partum haemorrhage (PPH, blood loss of >1000 cc during and after delivery), with about 146.000 deaths annually [1]. Although this is largely due to deaths in developing countries, even in the Netherlands it is still in the top four of maternal deaths [2]. In this country the reported incidence of PPH is 5% in the secondary care, affecting about 10.000 women annually [3]. In the Lemmon trial (2004-2006), in which a large part of Dutch hospitals participated, all severe maternal morbidity was documented and PPH in absolute number is the major factor contributing to severe maternal morbidity [4]. Causes such as substandard care are frequently reported in the international literature. In a study in France, suboptimal care factors were found in 38% of women with PPH > 1500 cc and in 70% of women who died of PPH. In audits, performed in PPH cases of the Lemmon trial, suboptimal care factors were also common in the Dutch population (unpublished data). A logical assumption is that these factors can be overcome by the use of evidence based guidelines. The Dutch Society of Obstetrics and Gynaecology (NVOG) developed and disseminated an evidence-based guideline about PPH, in which the best scientific evidence is summarized [5]. Furthermore, in 2003 the MOET course (Managing Obstetric Emergencies and Trauma), an ATLS-based course of the RCOG, translated to the Dutch situation, was introduced, in which stepwise and practical instructions to prevent PPH were given [6]. However, without tailor-made implementation, in general large gaps exist between best evidence as described in guidelines and daily practice [7,8]. This also applies to PPH. Both the dissemination of the evidence-based guideline about PPH and the MOET course did not lead to a reduction in PPH: nationwide the incidence of PPH was 3.8% in 2003 and 5.2% in 2006 [3]. An incomplete implementation of both the NVOG-guideline and the MOET-instructions is expected.

## Methods/Design

### Objectives

The first objective of this study is to asses to what extent the NVOG guidelines and the MOET-instructions have been implemented in current care in the Dutch practice. The second objective is to study barriers and facilitators for guideline adherence. Finally based on these findings a tailored implementation strategy will be developed and tested on effects, experiences and costs.

### Design and study population described per step

### Objective 1

To assess the actual care of Dutch gynaecologists and midwives for patients at high risk for PPH (actual care study).

### Design

#### Developing quality indicators

Before actual care can be measured, quality indicators have to be developed regarding the process, structure and outcome of care. These indicators have to be based on the key recommendations from the NVOG guideline on PPH and the MOET-instructions. The indicator development will be performed according to the RAND-modified Delphi method [9]. First the key recommendations from the NVOG guideline on PPH and the MOET-instructions will be extracted and relevant indicators from international literature will be added. Subsequently, the relevance of all these key recommendations will be tested in two rounds among an independent panel about 15 experts consisting of guideline writers, Dutch MOET board members and instructors, NVOG-members of the subcommittee Implementation and Quality, gynaecologists, haematologist and anaesthesiologist. In the first round the gathered recommendations will be edited in a written questionnaires for the expert panel where the experts are asked to score the key-recommendations on a 9-point Likert scale ranging from 1 = not relevant to 9 = extremely relevant, with respect to their impact on both 'health gain' and 'overall efficacy. In addition, a top-5 ranking of recommendations is asked in which they consider 'most important' and 'representative' to assess the quality of clinical performance. In this round the experts have the possibility to provide comments and add additional items as well. Of the returned questionnaires, the median scores on 'health gain' and 'overall efficacy' are calculated per recommendation and are rated valid if they match the criteria described by Campbell [10]. Secondly, based on the top-5 ranking of recommendations, a list with scores reflecting the weight that experts assigned to each recommendation will be created. In a second round, in a consensus meeting with all the experts these listings will be used as feedback. During this meeting this feedback will be discussed and the former rankings will be reconsidered with the aim to reach consensus about the most important recommendations to assess the quality of clinical performance regarding the adherence to the NVOG guideline on PPH and the MOET-instructions. The selected key recommendations will be operationalized in measurable elements.

#### The practical measurement of actual care

In an observational multi-centre study, actual care will be measured by video monitoring the third stage of delivery and a medical record search among 320 high risk patients for PPH in 20 hospitals. In all participating clinics, all delivery rooms will be set up with a digital camera. In order to avoid anxiety, bias or refusal of participation among the care-givers, it is made clear that none of the direct colleagues or patients will be able to see the images and that these images cannot be claimed by the patient in case of an adverse outcome. The images will be analyzed by the researcher and in a random selected subset by one of the project leaders to assess the extent of adherence to the developed quality indicators. Additional information for indicator adherence will be searched in the medical records of the videotaped patients. In this manner, deviations from the indicators can be outlined. This study will provide us with reliable information about current practice in the Netherlands.

### Study population

#### Hospitals

In order to obtain a representative view on the actual care in the Netherlands, 20 hospitals of different regions will participate in this trial including 4 academical, 8 non academical teaching and 8 non academical-non teaching hospitals. The study is set in a Dutch Perinatal Research Consortium, in which all the participating clinics collaborate.

#### Patients

All patients 18 years and older with a higher risk for PPH who will deliver in one of the participating hospitals can be included (16 patients per hospital). This will include women with PPH in a previous delivery, multiple pregnancy, polyhydramnion, chorio-amnionitis, uterus myomatosus, grande multiparity, long delivery, clotting disorders or thrombocytopenia (HELLP). Since asking permission during the delivery is difficult and the higher risk of PPH can develop during delivery, all women who visit the antenatal clinic will be asked in advance by research nurses to participate. Informed consent will be asked for filming the third stage of labour and for reviewing these images by a third party; the researcher. The group who declines will be asked permission to study their medical record. In this way, besides the total incidence of PPH in the study period, the incidence of PPH in women who participate and those who do not participate can be recorded

### Objective 2

To detect barriers and facilitators amongst professionals involved in the implementation of the NVOG-guideline on PPH and the MOET-instructions and patients (barriers and facilitators study).

### Design

A qualitative study will be performed with the aim to discover factors in detail that are "pro" or "contra" adhering to the developed PPH-indicators. This will be performed by focus group interviews among groups of different involved professionals (gynaecologists, midwives and gynaecologists in training) and experienced patients. The interviewer will explore the following categories of influencing factors: features of the guidelines itself; features of the target group of professionals who should use the recommendations; features of patients who have to accept or contribute to using the recommendations; features of the social setting and social network (e.g. colleagues of the involved professionals); features of the organizational, economic, and administrative context. Subsequently, to assess the 'prevalence' of the factors mentioned in the focus group interviews, a survey with questionnaires will be performed among all Dutch gynaecologists and midwives. They will receive a web-based questionnaire by e-mail. The data will be gathered in an electronic database.

### Study population

To select the members of the focus groups, we will contact the national professional associations of respectively gynaecologists and midwives and request nominations for opinion leaders and the professionals participating in the actual care study. The different focus groups will consist of respectively 10 gynaecologists, 10 midwives, 10 gynaecologists in training and 10 patients. To select the participants for the survey with questionnaires, we will contact the national professional associations of respectively gynaecologists and midwives and request e-mail addresses.

### Objective 3

Development and testing an implementation strategy in terms of effectiveness, experiences of participants, process and costs. (feasibility study):

### Design

Based on the results of step 1 and 2, a tailored implementation strategy will be developed to increase the adherence to the recommendations. Because different barriers at different levels are expected, it is very likely that a strategy with different implementation elements directed at both professional and organizational level will be developed. At this moment, we have some hypotheses about expecting limitations in actual care. At the level of the guideline/MOET-instructions itself we think that the guideline can be more specific; the description of the desired care is not detailed enough. The desired care can be described in a detailed and structural manor by the development of "bundles". These bundles are defined as a group of interventions related to a disease process that, when executed together, results in better outcome than when implemented individually. A second limitation could be a delayed time interval between events and taken actions, (right actions taken too slowly due to individual decisions or organizational factors). The solution could also be describing the desired care in bundles in the guideline and on organizational level an improvement process can be undertaken if the exact problem can be identified for example; multidisciplinary clear agreements. At the level of the professionals, a lack of knowledge/insight in own performance can be an impending factor. The installed monitors could be used to constant audit and feedback their performance, both individual and in review conferences and team training could be an intervention. However, due to uncertainty of existing barriers and facilitators the strategy can not be worked out in detail now.

### Intervention

The tailored improvement strategy will be implemented and evaluated in a feasibility study in nine months. The study will be performed in 4 hospitals (also participating in the actual care study) and consists of three evaluations:

a) To obtain an indication of the effect of the implementation strategy, the adherence to the developed indicators will be measured before (= actual care study) and after the introduction of the newly developed strategy, using videotaping and a medical record search (see step 1b). For the after-measurement we will include about 100 patients.

b) A process evaluation will be performed to study the experiences of the clinicians and patients with the changed care and also to study the extent by which clinicians and eventually patients use the elements of the strategies and their experiences (e.g. satisfaction and feasibility) with these elements. To achieve this, process information will be gathered in a qualitative study in the 4 participating hospitals and individual interviews will take place among the different involved gynaecologists, midwives and patients.

c) A cost analysis of the tested implementation strategy will take place. The perspective of this analysis will be the health care perspective. The costs of the implementation strategy will be estimated by an Activity Based Costing approach focusing on activities performed to implement the NVOG-guideline on PPH and the MOET-instructions, with the costs accumulated at the activity level(s) of the health care implementation processes. The costs of implementation of the guidelines and consolidation consist of personnel and material costs. The input of resources will be assessed by collecting volumes of consumed resources and multiplying these by the price of each resource unit. The prices of each resource unit will be based on standard costs, market prices or self-determined costs [11]. In the analysis, the implementation costs will be related to the difference in percentage of patients treated according to the guideline indicators in the situation before and after the implementation of the NVOG-guideline on PPH and the MOET-instructions

### Study population

To study the feasibility, four hospitals and their respective gynaecologists and midwives will participate in two different regions in the Netherlands. In each region, an academic hospital and a non-academic hospital will participate. The inclusion criteria and course of management for the 100 patients in the 'after-measurement' will be the same as in the actual care study. For the process evaluation all gynaecologists and midwives of the included patients are asked to participate.

### Outcome measures

#### Actual care study

The primary outcome measure is the adherence to the quality indicators (derived from the NVOG guideline on PPH and the MOET-instructions). The secondary outcome measure is outcome of care (e.g. the incidence of PPH).

#### Barrier and facilitator study

The main outcome is the different types and frequency of found barriers and facilitators for implementation of the NVOG guideline and MOET-instructions regarding gynaecologists, midwifes and patients.

#### Feasibility study

To get an indication of the effectiveness of the strategy the primary outcome measure is the adherence to developed quality indicators. Other outcome measures are the experiences of the participants (both professionals and patients) with the elements of the strategy and with the changed care. Also the cost of the tested strategy will be measured.

### Statistical issues

#### Sample size calculation actual care study and feasibility of recruitment

Assuming accordance with the guidelines of 50%, with an alpha of 0.05, a precision of the estimation of 0.075, 171 patients have to be included. However, taking clustering of data within clinicians into account, this number has to be multiplied by the design effect. With 5 patients per clinician and an intraclustercorrelation of 0.20 this effect is 1.8. So the minimum number of patients that have to be included is 1.8 × 171 = 308 patients. In order to compensate for lost to follow-up or incomplete data, 320 women have to be included in 20 hospitals in 6-9 months.

### Data analysis

#### Analysis of the actual care study

To analyse actual care, frequencies of adherence per quality indicator will be calculated. Furthermore, the variation in this care between the different hospitals will be calculated.

#### Analysis of the barriers and facilitators

The barriers and facilitators mentioned in the focus group interviews with professionals and patients will be qualitatively analysed, using the qualitative software package (Atlas). In the quantification of these barriers, frequencies of found barriers and facilitators will be calculated.

#### Analysis of the feasibility study

In the effectuation the proportion of patients that are treated in accordance with the guidelines (analysed on the basis of the indicator set) before and after implementation of the guidelines will be established. Both univariate and multivariate (multi-level) analyses are performed to demonstrate the effect of the implementation strategy in increasing the proportion of patients who are treated according to the guidelines. In addition, for the process evaluation, frequencies are used to asses the experiences of the professionals and patients to the implementation programme elements. Analyses of the costs of the implementation strategy will be conducted by multiplying the volumes of consumed resources with the price of each resource unit.

### Ethical considerations

The study protocol has been presented to the Medical Ethical Committee (CMO) of the region Arnhem and Nijmegen (ABR no. NL25975.091.08). Ethical approval was not necessary. The protocol is registered in the ClinicalTrials.gov register (NCT00928863)

## Discussion

This study addresses an important problem because PPH is currently the major cause of severe maternal morbidity in the Netherlands. Many different factors determine the action that is taken in case of more than average blood loss or once a real PPH sets in. Insight into these factors is of great importance in order to know what kind of activities should be developed to prevent PPH by implementing the NVOG-guideline and MOET-instructions. In literature, the following facilitators and barriers are distinguished: features of the innovations itself, of the target group of professionals who should use the innovation, of patients who have to accept or contribute to the innovation, of the social setting and network and of the organisational, economic and administrative context [12]. To our knowledge the proposed study is the first study on barriers and the development and testing of a tailored implementation strategy for acute care situations in the obstetrics. A randomized controlled study is the next step to measure the effectiveness of the implementation of the obtained strategy if the result of this study is that the strategy is feasible in practice, can be implemented with low costs and indicates to be effective. Ultimately, the generated knowledge and understanding of the implementation process can be used to implement guidelines in different countries with similar problems and hopefully lead to a worldwide reduction of the incidence of PPH

## Abbreviations

HELLP: Haemolysis Elevated Liver enzymes Low platelets; RCOG: Royal College Obstetric Gynaecology; ATLS: Advance Trauma Life Support.

## Competing interests

The authors declare that they have no competing interests.

## Authors' contributions

HS and RH were involved in conception and design of the study. MW, HS and RH drafted the manuscript. All authors mentioned in the manuscript are member of the Fluxim study group. They participated in the design of the study during several meetings. All authors edited the manuscript and read and approved the final manuscript.

## Pre-publication history

The pre-publication history for this paper can be accessed here:

http://www.biomedcentral.com/1471-2393/10/5/prepub
